# Deep Learning and Simulation for the Estimation of Red Blood Cell Flux With Optical Coherence Tomography

**DOI:** 10.3389/fnins.2022.835773

**Published:** 2022-02-17

**Authors:** Sabina Stefan, Anna Kim, Paul J. Marchand, Frederic Lesage, Jonghwan Lee

**Affiliations:** ^1^School of Engineering, Brown University, Providence, RI, United States; ^2^Department of Neuroscience, Brown University, Providence, RI, United States; ^3^Department of Electrical Engineering, École Polytechnique de Montréal, Montréal, QC, Canada; ^4^Carney Institute for Brain Science, Brown University, Providence, RI, United States

**Keywords:** RBC flux, deep learning, simulation, capillary flow, microvascular network

## Abstract

We present a deep learning and simulation-based method to measure cortical capillary red blood cell (RBC) flux using Optical Coherence Tomography (OCT). This method is more accurate than the traditional peak-counting method and avoids any user parametrization, such as a threshold choice. We used data that was simultaneously acquired using OCT and two-photon microscopy to uncover the distribution of parameters governing the height, width, and inter-peak time of peaks in OCT intensity associated with the passage of RBCs. This allowed us to simulate thousands of time-series examples for different flux values and signal-to-noise ratios, which we then used to train a 1D convolutional neural network (CNN). The trained CNN enabled robust measurement of RBC flux across the entire network of hundreds of capillaries.

## Introduction

The microvascular bed is integral to the delivery of nutrients and oxygen to cerebral tissue, and its dysfunction is implicated in a number of pathologies. To understand how various experimental conditions impact microvascular flow, it is critical to be able to study a large number of capillaries considering the heterogeneity of flow in capillaries. The ability to study blood flow in a network of interconnected capillaries makes it possible to test a wide range of scientific hypotheses on how microvascular flow is regulated in the brain in health and disease. For example, microvascular flow has been studied in relation to neurovascular coupling (Rungta et al., 2018), stroke (Schaffer et al., 2006) and Alzheimer’s disease (Gutiérrez-Jiménez et al., 2018).

However, it is not currently feasible to accomplish high-throughput flow measurements using two-photon excitation fluorescence microscopy (TPEF), the gold standard for quantitative microvascular imaging (Kleinfeld et al., 1998; Nelson et al., 2020; Shaw et al., 2021). As an alternative, optical coherence tomography (OCT) has shown promising results for the quantification of blood flow, as demonstrated by Doppler OCT for imaging arterioles and venules (Munce et al., 2010; Srinivasan et al., 2010). Doppler OCT has also shown some promise in quantifying red blood cell (RBC) speed (Tang et al., 2017), although RBC flux may be more physiologically relevant as it more closely correlates with oxygenation (Li et al., 2019). However, in comparison to larger vessels, the dynamics of blood flow differ greatly in capillaries which are typically smaller than 10 μm and in which RBCs flow discretely in single file, making it difficult to apply Doppler OCT to determine RBC flux. This is because the fast A-scan rate defining Doppler OCT results in only 1–2 RBC passages being observed during a reasonable acquisition period, which is insufficient to determine RBC flux. Instead, OCT has been leveraged to quantify RBC flux in large networks of capillaries, based on the observation that a passage of a RBC through a voxel causes a transient increase in intensity (Ren et al., 2012; Lee et al., 2013). The standard approach is to count the number of peaks observed over a length of time using some peak-finding algorithm, commonly based on a threshold: i.e., peaks over this threshold are considered to be peaks resulting from RBC passages (Li et al., 2016). However, this approach is sensitive to noise, requires user parametrization, and has been shown to underestimate RBC flux at high flux values (over 80 RBC/s) (Marchand et al., 2020). Often, it is difficult to manually distinguish noise from legitimate RBC passages, making it difficult to assess whether a peak-counting algorithm is working correctly or whether the threshold is appropriate.

To address this issue, we aimed to develop a robust and accurate method of determining RBC flux from OCT data which does not require any user parametrization. We exploited deep learning to extract relevant features from OCT time-series data using a 1D convolutional neural network (CNN). To train the network, we collected RBC passage data using OCT and TPEF simultaneously, where the TPEF data provided the true flux value. We probed the CNN to understand which features were important for classification of RBC flux, and then incorporated these results to simulate many OCT time-traces for different RBC flux and velocity values. This enabled us to produce hundreds of thousands of training examples, which is especially important for low and high flux values which are underrepresented in real data. The result is a validated method for simulating OCT time-series data, and a CNN that can be implemented to obtain accurate and robust estimations of RBC flux in large networks of capillaries.

## Dataset

As described by Marchand et al. (2020), a multi-modal platform was designed for simultaneous *in vivo* imaging using OCT and TPEF. TPEF imaging was performed at 920 nm and FITC fluorescence was collected through an emission filtered at 520 nm. An objective lens with magnification of 10× (Mitutoyo) was used to provide a lateral resolution <3 μm for TPEF imaging, and 3.5 μm for OCT. The light source of the OCT system operated at 1300 nm (LS2000C, Thorlabs) with an axial resolution of ∼ 3.8 μm in animal tissue.

For *in vivo* imaging of the cerebral cortices of C57BL/6 mice, we performed craniotomies with approval from the ethics committee of the research center of the Montreal Heart Institute. For the craniotomy performed at Brown University, experimental procedures were reviewed and approved by the Institutional Animal Care and Use Committee (IACUC) of Brown University and Rhode Island Hospital. Cranial windows were installed to gain optical access to the cerebral cortex, according to the widely adopted protocol described by Mostany and Portera-Cailliau (2008).

To obtain the simultaneous time-courses using OCT and TPEF, we performed imaging in the awake state. Firstly, we selected a capillary through the TPEF channel, since only a single capillary can be analyzed at a time using TPEF. Line-scanning was performed at a rate of 800 Hz along the longitudinal axis of the capillary for approximately 60 s along a line of 11.7 μm. At the same time, B-scans were acquired using the OCT to capture the intensity time-course of the same vessel. These B-scans were repeated over a time of 0.768 s at a rate of 665 Hz, resulting in a temporal resolution of 1.5 ms. Each B-scan consisted of 60 × 2048 pixels, spanning 100 μm laterally, resulting in a lateral pixel size of 1.7 μm. The TPEF time-course was then downsampled to the same frequency as the OCT. Overall, 9441 time-courses of RBC passages were obtained from a total of 3 mice and 119 capillaries.

We performed additional OCT imaging to demonstrate our method on a large network of capillaries, which we performed using a commercial SD-OCT system (Telesto III, Thorlabs) with center wavelength of 1310 nm and bandwidth of 170 nm. This imaging was performed on one C57BL/6 mouse under 2% isoflurane anesthesia. As before, we used a 10× objective lens (Mitutoyo) with transverse resolution of 3.5 μm. To image the network of capillaries, we scanned a region with a field of view of 256 × 256 × 70 pixels (x, y, z) corresponding to a size of 768 × 768 × 245 μm (x, y, z). We did so by repeatedly performing B-scans at a rate of 714 Hz at each y position for over a time of 0.72 s, yielding a temporal resolution of 1.4 ms. We believe these imaging parameters to be appropriate for the intended purpose of measuring RBC flux in a network of capillaries, because the resolution is sufficient to observe individual capillaries and individual passages of RBCs, yet also balances the trade-off between resolution, dataset size, and acquisition time.

## Training 1D Convolutional Neural Network on Experimental Data

We acquired 9441 time-traces of RBC passages using OCT and TPEF as described by Marchand et al. (2020), consisting of 512 time-points at a temporal resolution of 1.5 ms. TPEF is often regarded as the gold standard for RBC flux measurements, since the TPEF time-traces exhibit high signal-to-noise ratio and are not plagued by speckle noise like the OCT data, allowing us to obtain the “ground truth” for the RBC flux. To confirm the higher signal-to-noise ratio, we determined the standard deviation of the normalized OCT and TPEF time-courses, and found a statistically higher signal-to-noise ratio as defined by the coefficient of variation for the TPEF time-courses (*p* < 0.001; paired *t*-test). Thus, we trained a 1D CNN, using the OCT time-traces as the training data and the RBC flux values obtained using TPEF as the labels. We augmented this dataset by reversing the time of the time-traces, and then split the augmented dataset into training, validation and testing with proportions of 70, 20, and 10%, respectively.

We adopted the state-of-the-art architecture for time-series classification, InceptionTime (Ismail Fawaz et al., 2020). The CNN consists of an input layer of size 512 (corresponding to 0.75 ms), 3 inception modules, a global averaging layer, and a fully-connected layer which results in 2 outputs: the RBC flux prediction and its associated uncertainty. The inception module consists of a bottleneck layer, which feeds into 3 convolutional layers of kernel size of 10, 20, and 40. At the same time, the input of the inception module is also fed through a max-pooling layer of kernel size 3, and then a bottleneck layer. The output of the bottleneck layer and the convolutional layers are concatenated channel-wise and form the input of the next inception module.

We designed our CNN to provide an estimate of uncertainty in its prediction, which serves as a signal that a prediction may not be trusted. This is important because there is ambiguity in what constitutes the passage of a RBC, even when assessing the data manually. To provide a measure of uncertainty we adapted the loss function as shown in Eq. 1 to maximize the probability of a given prediction y, assuming this prediction is derived from a normal probability distribution with mean equal to the ground truth, G, and standard deviation, σ. The standard deviation, which is learned by the network, thus serves as an indicator of uncertainty in the predicted value. The network thus provides a continuous prediction for RBC flux, along with its continuous predicted uncertainty. We trained our network using the Adam optimizer, with a learning rate of 8e-4 for 50 epochs, and a gradient threshold of 500 which was necessary given our adapted loss function.


(1) $$\mathrm{loss}=-\log\,\left(\frac{1}{\sigma \,\sqrt{2\,\pi }}\,\exp\left(\frac{{\left(G-\hat{y}\right)}^{2}}{2\,\sigma^{2}}\right)\right)$$

**FIGURE 1:**
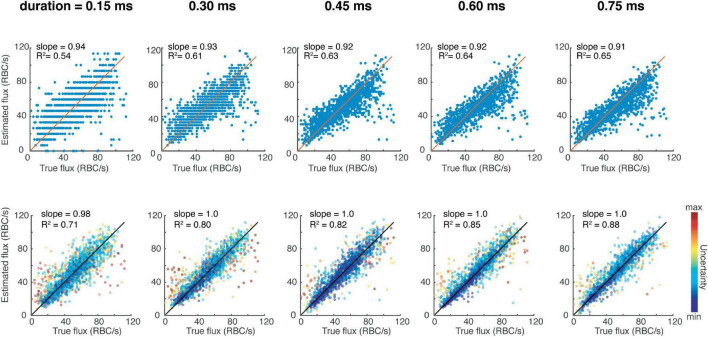
Red blood cell fluxestimation accuracy as a function of acquisition time for the traditional peak counting method **(top row)** versus the presented deep learning-based method **(bottom row)**.

We tested the performance of the CNN on the test data, which was unseen to the CNN during training, and observed more accurate results compared to peak-counting as assessed by the slope being closer to 1, and the *R*^2^ value higher (Figure 1). Therefore, the CNN provides the possibility to filter the predictions to include data with the highest confidence. We then tested the same network architecture trained on different input sizes to understand the trade-off between acquisition time and accuracy (Figure 1). It is important to note that increasing the acquisition time does improve the *R*^2^ value of the peak-finding estimations, the slope is consistently less than 1, indicating a systematic bias against high-flux capillaries. On the other hand, the CNN can provide accurate predictions even for short acquisition times. We also tested this loss function in comparison to a conventional mean-squared loss, which does not provide a measure of uncertainty in its prediction. To do so, we trained a separate CNN with this mean-squared loss to perform regression on the same dataset as previously, and found that these results were still better than traditional peak-fitting (slope = 0.98, *R*^2^ = 0.85), although it is not possible to weight predictions based on uncertainty like afforded by the loss function described in Eq. 1.

## Simulating Optical Coherence Tomography Time-Series Data

**FIGURE 2:**
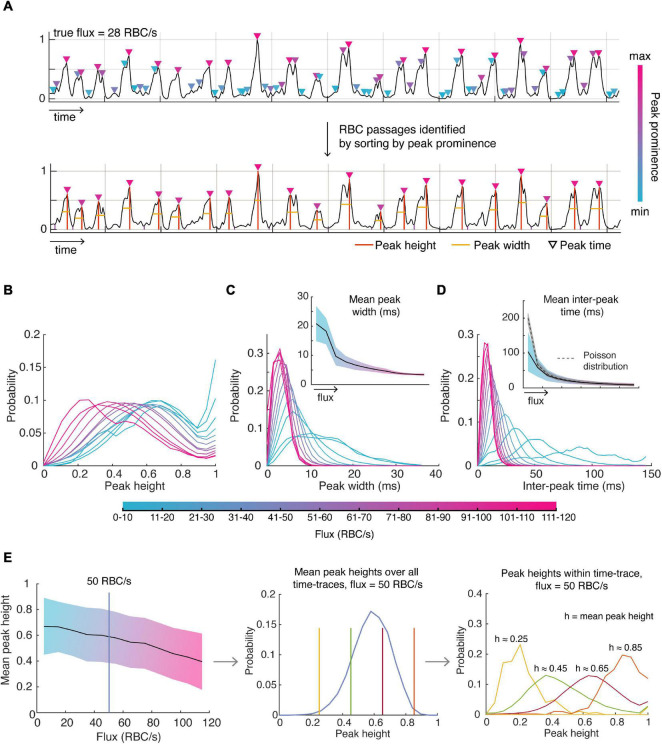
Detecting RBC passages from OCT time-series data and characterizing the distributions of parameters describing these RBC passages. **(A)** RBC passages were identified using the known value of flux (obtained by TPEF) and sorting peaks based on peak prominence. Orange lines describe peak position and heights, and the yellow line is the full width half maximum (FWHM), describing the width. **(B)** The distribution of all peak heights **(C)** peak widths and **(D)** inter-peak times for varying ranges of flux values. **(E)** Mean peak height decreases with increasing flux (left). Mean peak height over all time-traces for each flux category is described by a beta distribution (middle) as shown using 50 RBC/s as an example, and the peak heights within a time-trace given the mean peak height are also described by the beta distribution (right). Shaded areas show the standard deviation about the mean.

A significant benefit of knowing the ground truth for flux values is to enable determination of the underlying distributions of parameters describing the OCT time-series data: the shape, width, height and spacing of the peaks. We firstly identified likely RBC passages within a time-trace by identifying all peaks, sorting these peaks by peak prominence, and selecting the number of peaks corresponding to the true flux in order of descending peak prominence (Figure 2A). We used these peaks to assess the distributions of the parameters. We found that normalized time-traces with flux values between 20 and 80 RBC/s had peaks which had mean heights closer to 1 after normalization of each time-trace (Figure 2B), indicating that these peaks are likely to be detected by the traditional threshold-based methods. However, time-traces with higher flux values tend to have more peaks with smaller heights which may explain why the traditional methods tend to severely underestimate high RBC flux passages. This highlights the inherent difficulty in choosing an appropriate threshold to be sensitive to capture RBC passages in high flux capillaries while minimizing false positives in low flux capillaries. After having characterized the distribution of mean peak heights for each flux, we sought to describe the distribution of peak heights for a given flux and mean peak height within each time-trace, as shown in Figure 2E. For example, we find the mean peak height across all time-traces for a given flux is described by a beta distribution. Now, for a given mean peak height and flux, the height of intensity peaks within those time-traces are also described by a beta distribution, but with different parameters. Thus, for each flux and mean peak height, there is a unique beta distribution describing the peak heights within each time-trace, which is also true for peak width.

As expected, lower flux values are associated with larger peak widths considering the relationship between peak width and RBC speed (Figure 2C). The mean peak width for each flux value was well captured by the gamma distribution. For a given mean width, the gamma distribution also described well the distribution of peak widths within a time-trace. We also verify that the standard deviation of the average peak width decreases with increasing flux since large flux values can only be accomplished with higher RBC speeds (due to the finite size of the RBC) whereas low flux can be accomplished with low or high-speed RBCs. We also assessed the relationship between mean peak width and peak width variability, especially in the context of RBC speed, which was approximated as the reciprocal of the peak width. For example, we tested whether low RBC speed time-traces (i.e., high mean peak width) was also associated with greater variability in RBC speed, but we did not observe any relationships between mean RBC speed and RBC speed variability.

Next, we looked to describe the spacing between RBC peaks, or the inter-peak time (Figure 2D). As expected, the mean inter-peak time is inversely proportional to the flux – only the lowest flux category (<10 RBC/s) vastly deviated from this relationship by exhibiting lower mean inter-peak time than expected, likely an artifact due to the small number of RBCs observed during duration of each time-trace. We expected the distribution of inter-peak distances to follow a Poisson distribution with mean equal to the mean inter-peak distance. However, we found that the experimental standard deviation was greater for low flux values than predicted by a Poisson distribution (i.e., greater than the square root of the mean), only approaching a Poisson distribution for high flux values.

Having characterized the parameter distributions describing how RBC passages appear in OCT data across all flux ranges, we were able to use this information to simulate RBC passages computationally for OCT imaging specifications similar to those described in the section Dataset. This will allow us to produce many more training examples of low and high flux time-traces for further training of our CNN, which were underrepresented in the experimental data. This also allowed us to produce many more training examples of rare but physiological instances, such as low-flux, high-speed time-traces. We first replicated our experimental data by simulating RBC passages as Gaussian pulses with identical mean height, width, and inter-peak distances. We then had the CNN of Figure 1 predict flux values from this set of noiseless, simulated training time-traces. Importantly, we discovered that the CNN overestimated low flux values and underestimated high flux values, implying that noise is an essential feature for the prediction of RBC flux.

## Improving Optical Coherence Tomography Time-Series Simulations

We hypothesized that OCT-specific noise and slow-varying components are critical features that were omitted in the simple simulation above. Thus, we modeled OCT-specific noise and added the noise as well as slow-varying components, and then tested if the improved simulation improves prediction by the CNN.

We modeled OCT-specific noise as described in Uribe-Patarroyo et al. (2020). Here noise, N, is a random variable described by a zero-mean complex circular Gaussian distribution, which will be added to the signal, S, like so,


(2) $$I=S+N^{2}+2\,\sqrt{S}\,\left|N\right|\,\cos\phi$$

**FIGURE 3:**
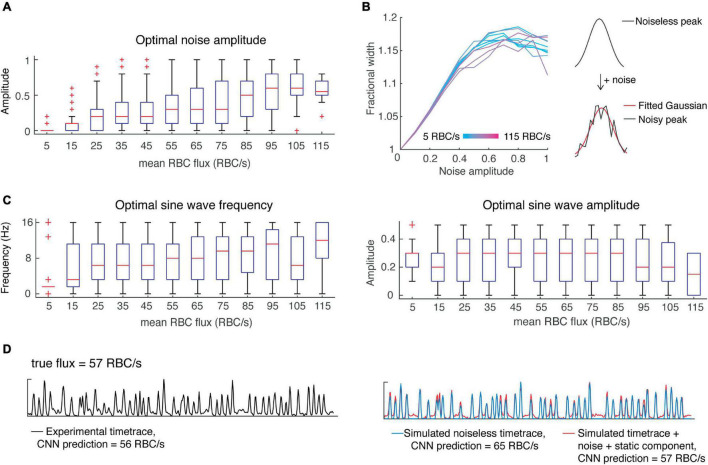
Incorporating noise and a slow-varying component improves predictions of CNN on simulated data. **(A)** Optimal noise amplitudes to maximize CNN prediction accuracy as a function of flux. **(B)** Including noise has the effect of increasing peak width as a function of the noise amplitude, reaching a plateau at around 17%. **(C)** Low flux value time-traces are predicted with greater accuracy when including a slowly-varying component with low frequency, whereas all other flux values show greater variability in the optimal parameters of the slow-varying component. **(D)** An example showing how incorporating noise and a slow-varying component results in more accurate CNN predictions.

Where φ is the relative phase between S and N and is modeled by a uniform distribution. Before incorporating noise into our simulations, we first tested whether the noise would affect the width of the simulated peaks. In detail, we added noise to the noiseless, simulated signals described above, while varying the amplitude of noise to achieve a range of signal-to-noise ratios from 1 to 10. We applied a gaussian filter with sigma = 1.5 ms, while taking into account the effect of filtering on peak width. As expected, increasing noise resulted in an increased peak width, eventually reaching a plateau (Figure 3B). Furthermore, the fractional increase in peak width was independent of the original peak width. This corruption of peak width by noise may help explain the inaccuracy of estimation of RBC speed using OCT time-traces (Marchand et al., 2020).

Next, we wanted to investigate whether noise amplitude was flux-dependent in the experimental data, and whether adding noise could improve the CNN predictions of our simulated data – i.e., is noise a critical feature needed for the classification of flux by our CNN? To answer these questions, we performed a grid search over different noise amplitudes (SNR ranging from 1 to 10) while correcting for the effect of noise on peak width. We found the optimal noise amplitude for each time-trace and assessed these results as a function of flux (Figure 3A). For high flux values (>100 RBC/s), we found better predictions were obtained with greater noise amplitudes, whereas for mid-range fluxes (∼50–100 RBC/s), a wide range of noise amplitudes yielded optimal predictions. On the other hand, predictions of low flux values were almost always hindered by the addition of noise. This result suggests that there is a positive correlation between RBC flux and noise, which should be taken into account when designing algorithms for the simulation of OCT time-traces and the estimation of RBC flux.

We simulated noisy data using the average optimal noise amplitude for each flux range and verified that the number of peaks detected (using the previously determined peak prominence) was the same before and after the addition of noise. The CNN predictions from this noisy simulated data were improved in comparison to those from the noiseless data, except for very low flux values (<15 RBC/s) which were overestimated.

In addition to noise, OCT time-series can include a slow-varying component due to static scattering (Srinivasan et al., 2012). Therefore, we tested whether including a slow-varying component would improve the CNN predictions in a similar manner. We simply modeled this component as a sine wave with unknown amplitude and frequency and added it to the noisy simulated signals. Like above, we performed a grid search to determine the optimal parameters as a function of flux (Figure 3C). Interestingly, the predictions for low flux values (<15 RBC/s) were greatly improved by incorporating a slow-varying component, in the form of a sine wave with frequency 1.5 Hz and amplitude of 0.3. But predictions for higher flux values were improved by a much lesser extent. Based on these findings, we again modeled RBC passages as Gaussian pulses with height, width and inter-peak time using the uniform distribution as well as the distributions found above. To secure a sufficient number of training samples of rare instances, such as low flux capillaries with high RBC speed, we sampled parameters from uniform distributions describing the mean height, width, and inter-peak time. For example, for a given flux, we sampled from a uniform distribution describing mean peak height. Now given this mean peak height, we sampled from the experimentally-determined beta distribution to obtain the peak heights of all peaks within the time-trace (Figure 2E). We followed a similar procedure for peak widths and inter-peak times, which have variability within each time-trace as described by gamma distributions. We found these parameters to be independent, allowing us to sample from each distribution independently. We then added noise (Eq. 2) with varying amplitude from 0 to 1, and sine waves with frequencies from 0 to 16 Hz and amplitudes from 0 to 0.5. We incorporated these elements by sampling from the distributions describing noise amplitude and static components as a function of flux (Figure 3).

## Training of the Convolutional Neural Network on Simulated Data

Having simulated OCT time-traces of RBC passages much more realistically by adding the modeled noise and slow-varying components, we further trained the CNN of Figure 1 with these realistic simulated signals, the number of which is much larger than the experimental data. We added these simulated signals to the experimental signals, producing a training sample of 10,000 training examples for each flux category, resulting in 1,20,000 total training samples.

**FIGURE 4:**
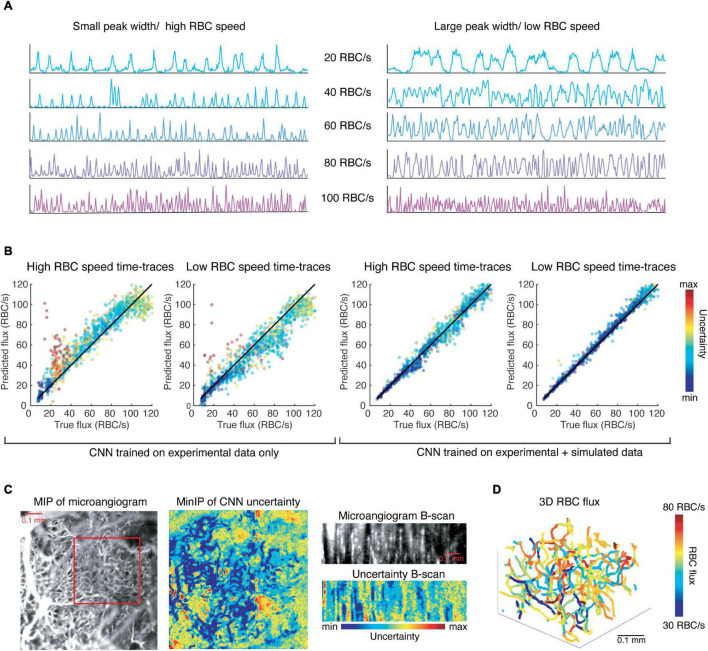
Convolutional neural network trained on simulated data is more robust to different RBC speeds (peak widths) than the CNN trained only on experimental data and can be utilized to extract RBC flux in a network of capillaries. **(A)** Simulated time-traces with the smallest (left) and largest (right) mean peak widths that were observed experimentally. **(B)** Results of the CNNs trained on experimental data and simulated data, respectively, with the input are time-traces like those shown in **(A)**. **(C)** The CNN predicts flux values with low uncertainty for voxels corresponding to capillaries. **(D)** The 3D RBC flux of the capillaries shown in the red square in **(C)**.

With this data, we continued training the CNN described previously and found a modest improvement in the resulting accuracy as determined by testing the network on the experimental test data (*R*^2^ value increased from 0.88 to 0.91, slope = 1). However, this network is more robust to different noise levels, and different RBC speeds for any given flux value, as shown in Figure 4. The network trained solely on experimental data systematically overestimates fluxes of simulated time-traces with smaller peaks widths, which is correlated with higher RBC speed (Figure 4A), even when the RBC speed is within a physiological range. Similarly, this CNN underestimates flux from time-traces with larger peak widths than average (Figure 4B). Simulating time-traces allows for more balanced training data, which serves to eliminate this bias.

## Red Blood Cell Flux of Capillary Network

We applied our CNN to 4D data (x, y, z, t) of the murine cortical vasculature to estimate RBC flux in an interconnected network of capillaries, which was unseen to the CNN during training. The data was collected over a FOV of 0.768 mm × 0.768 mm × 0.25 mm (x, y, z), and for a duration of 716 ms with temporal resolution of 1.4 ms. As a sign of confidence in our method, we observed that voxels corresponding to capillaries were associated with lower uncertainty as predicted by the CNN (Figure 4C). We applied our previously-developed toolbox for the segmentation of OCT angiograms to obtain the centerlines of each vessel (Stefan and Lee, 2020). We then applied the CNN to the time-trace of each of the voxels within the centerline of a vessel and averaged the predicted flux values for the vessel while weighting the predictions by their associated uncertainties. Repeating this prediction and averaging for every vessel in a capillary network produced a map of RBC fluxes (Figure 4D). While Marchand et al. used a masking technique to identify the best voxel choice, we recentered the vessel skeleton using the algorithm described in Stefan and Lee (2020) to align the centerline with the voxels of lowest uncertainty.

## Discussion

It is useful to obtain high-throughput estimations of RBC flux, as it allows for the study of capillary network dynamics with greatly improved statistical power. OCT has been shown to be a promising tool for this purpose, considering that OCT can detect individual RBC passages due to the transient increases in intensity as the RBC passes through the imaging voxel. However, it is still unclear on how to identify these RBC passages from OCT time-traces. Basic peak-finding algorithms were an obvious starting point but have recently been shown to systematically underestimate high flux values.

By investigating time-traces obtained using OCT and TPEF simultaneously, we have uncovered the distributions governing the height, width and inter-peak time of those intensity peaks representing the passage of RBCs. Our findings reveal that time-traces of high flux capillaries tend to have lower mean peak height, which explains why the traditional peak-counting methods underestimate RBC flux for these capillaries. Our findings also indicate that time-traces of high flux capillaries tend to have greater noise, which in turn may impact peak detection and peak width measurement. Adding noise increased the peak width by up to 17%, which may contribute to error when estimating RBC speed. We also found that the incorporation of slowly-varying components was critical to the accurate prediction of RBC flux, in particular for low flux values, suggesting that this may be a common component of time-traces of low-flux capillaries. These insights may serve to form a foundation for how researchers may simulate OCT time-traces and also some of the challenges that need to be overcome. Subsequent studies may also study the effects of multiple-scattering, lateral resolution, and the orientation of the capillary (specifically, nearly-vertically oriented capillaries). Furthermore, we believe this work extends to other mammals considering that the variation of RBC size is small (Hawkey et al., 1991), capillaries are of similar diameter (Karbowski, 2011), and RBC velocity appears to be independent of body mass (Unekawa et al., 2010).

We utilized these findings and empirical distributions to simulate OCT time-traces for a wide range of RBC fluxes and speeds, as well as noise amplitudes. This novel simulation strategy which better mimics real OCT time-traces has enabled us to develop a more balanced training set than the one acquired experimentally as well as thousands of more training examples, thereby improving the robustness of the network. We make the code freely available for researchers to implement our trained CNN directly to their data without any user parametrization, as well as code for simulating OCT time-traces and training their own networks with any input size.

## Data Availability Statement

The raw data supporting the conclusions of this article will be made available by the authors, without undue reservation. Code and trained neural networks are available at this link: https://github.com/sstefan01/RBCflux.

## Ethics Statement

The animal study was reviewed and approved by Institutional Animal Care and Use Committee (IACUC) of Brown University.

## Author Contributions

SS performed the analyses, acquired experimental data, and wrote the manuscript. AK performed animal surgeries. PM and FL acquired the simultaneous TPEF and OCT data. JL consulted the results and supervised the project. All authors revised the manuscript and approved the submitted version.

## Conflict of Interest

The authors declare that the research was conducted in the absence of any commercial or financial relationships that could be construed as a potential conflict of interest.

## Publisher’s Note

All claims expressed in this article are solely those of the authors and do not necessarily represent those of their affiliated organizations, or those of the publisher, the editors and the reviewers. Any product that may be evaluated in this article, or claim that may be made by its manufacturer, is not guaranteed or endorsed by the publisher.

## References

[B1] Gutiérrez-Jiménez E. Angleys H. Rasmussen P. M. West M. J. Catalini L. Iversen N. K. (2018). Disturbances in the control of capillary flow in an aged APPswe/PS1ΔE9 model of Alzheimer’s disease. *Neurobiol. Aging* 62 82–94. 10.1016/j.neurobiolaging.2017.10.006 29131981

[B2] Hawkey C. M. Bennett P. M. Gascoyne S. C. Hart M. G. Kirkwood J. K. (1991). Erythrocyte size, number and haemoglobin content in vertebrates. *Br. J. Haematol.* 77 392–397. 10.1111/j.1365-2141.1991.tb08590.x 2012765

[B3] Ismail Fawaz H. Lucas B. Forestier G. Pelletier C. Schmidt D. F. Weber J. (2020). InceptionTime: finding AlexNet for time series classification. *Data Min. Knowl. Discov.* 34 1936–1962. 10.1007/s10618-020-00710-y

[B4] Karbowski J. (2011). Scaling of brain metabolism and blood flow in relation to capillary and neural scaling. *PLoS One* 6:e26709. 10.1371/journal.pone.0026709 22053202 PMC3203885

[B5] Kleinfeld D. Mitra P. P. Helmchen F. Denk W. (1998). Fluctuations and stimulus-induced changes in blood flow observed in individual capillaries in layers 2 through 4 of rat neocortex. *Proc. Natl. Acad. Sci. U. S. A.* 95 15741–15746. 10.1073/pnas.95.26.15741 9861040 PMC28114

[B6] Lee J. Wu W. Lesage F. Boas D. A. (2013). Multiple-capillary measurement of RBC speed, flux, and density with optical coherence tomography. *J. Cereb. Blood Flow Metab.* 33 1707–1710. 10.1038/jcbfm.2013.158 24022621 PMC3824190

[B7] Li B. Esipova T. V. Sencan I. Kılıç K. Fu B. Desjardins M. (2019). More homogeneous capillary flow and oxygenation in deeper cortical layers correlate with increased oxygen extraction. *Elife* 8:e42299. 10.7554/eLife.42299 31305237 PMC6636997

[B8] Li B. Lee J. Boas D. A. Lesage F. (2016). Contribution of low- and high-flux capillaries to slow hemodynamic fluctuations in the cerebral cortex of mice. *J. Cereb. Blood Flow Metab.* 36 1351–1356. 10.1177/0271678X16649195 27165011 PMC4976754

[B9] Marchand P. J. Lu X. Zhang C. Lesage F. (2020). Validation of red blood cell flux and velocity estimations based on optical coherence tomography intensity fluctuations. *Sci. Rep.* 10:19584. 10.1038/s41598-020-76774-z 33177606 PMC7658245

[B10] Mostany R. Portera-Cailliau C. (2008). A craniotomy surgery procedure for chronic brain imaging. *JoVE* 12:e680. 10.3791/680 19066562 PMC2582844

[B11] Munce N. R. Wright G. A. Mariampillai A. Standish B. A. Leung M. K. K. Tan L. (2010). Doppler optical coherence tomography for interventional cardiovascular guidance: in vivo feasibility and forward-viewing probe flow phantom demonstration. *J. Biomed. Opt.* 15:011103. 10.1117/1.3292007 20210429

[B12] Nelson A. R. Sagare M. A. Wang Y. Kisler K. Zhao Z. Zlokovic B. V. (2020). Channelrhodopsin excitation contracts brain pericytes and reduces blood flow in the aging mouse brain in vivo. *Front. Aging Neurosci.* 12:108. 10.3389/fnagi.2020.00108 32410982 PMC7201096

[B13] Ren H. Du C. Park K. Volkow N. D. Pan Y. (2012). Quantitative imaging of red blood cell velocity invivo using optical coherence Doppler tomography. *Appl. Phys. Lett.* 100:233702–2337024. 10.1063/1.4726115 22904572 PMC3382255

[B14] Rungta R. L. Chaigneau E. Osmanski B. F. Charpak S. (2018). Vascular compartmentalization of functional hyperemia from the synapse to the pia. *Neuron* 99:362–375.e4. 10.1016/j.neuron.2018.06.012 29937277 PMC6069674

[B15] Schaffer C. B. Friedman B. Nishimura N. Schroeder L. F. Tsai P. S. Ebner F. F. (2006). Two-photon imaging of cortical surface microvessels reveals a robust redistribution in blood flow after vascular occlusion. *PLoS Biol.* 4:e22. 10.1371/journal.pbio.0040022 16379497 PMC1324794

[B16] Shaw K. Bell L. Boyd K. Grijseels D. M. Clarke D. Bonnar O. (2021). Neurovascular coupling and oxygenation are decreased in hippocampus compared to neocortex because of microvascular differences. *Nat. Commun.* 12 1–16. 10.1038/s41467-021-23508-y 34045465 PMC8160329

[B17] Srinivasan V. J. Radhakrishnan H. Lo E. H. Mandeville E. T. Jiang J. Y. Barry S. (2012). OCT methods for capillary velocimetry. *Biomed. Opt. Express*. 3 612–629. 10.1364/boe.3.000612 22435106 PMC3296546

[B18] Srinivasan V. J. Sakadžiæ S. Gorczynska I. Ruvinskaya S. Wu W. Fujimoto J. G. (2010). Quantitative cerebral blood flow with optical coherence tomography. *Opt. Express* 18 2477–2494. 10.1364/oe.18.002477 20174075 PMC2837842

[B19] Stefan S. Lee J. (2020). Deep learning toolbox for automated enhancement, segmentation, and graphing of cortical optical coherence tomography microangiograms. *Biomed. Opt. Express* 11:7325. 10.1364/boe.405763 33409000 PMC7747889

[B20] Tang J. Erdener S. E. Fu B. Boas D. A. (2017). Capillary red blood cell velocimetry by phase-resolved optical coherence tomography. *Opt. Lett.* 42 3976–3979. 10.1364/ol.42.003976 28957175 PMC5972360

[B21] Unekawa M. Tomita M. Tomita Y. Toriumi H. Miyaki K. Suzuki N. (2010). RBC velocities in single capillaries of mouse and rat brains are the same, despite 10-fold difference in body size. *Brain Res.* 1320 69–73. 10.1016/j.brainres.2010.01.032 20085754

[B22] Uribe-Patarroyo N. Post A. L. Ruiz-Lopera S. Faber D. J. Bouma B. E. (2020). Noise and bias in optical coherence tomography intensity signal decorrelation. *OSA Contin.* 3 709–741. 10.1364/osac.385431 34085035 PMC8171193

